# Relationship between serum osteocalcin level and carotid intima-media thickness in a metabolically healthy Chinese population

**DOI:** 10.1186/s12933-015-0245-9

**Published:** 2015-06-16

**Authors:** Yuqi Luo, Xiaojing Ma, Yaping Hao, Qin Xiong, Yiting Xu, Xiaoping Pan, Yuqian Bao, Weiping Jia

**Affiliations:** Department of Endocrinology and Metabolism, Shanghai Jiao Tong University Affiliated Sixth People’s Hospital; Shanghai Clinical Center for Diabetes; Shanghai Key Clinical Center for Metabolic Disease; Shanghai Diabetes Institute; Shanghai Key Laboratory of Diabetes Mellitus, 600 Yishan Road, Shanghai, 200233 China

**Keywords:** Osteocalcin, Carotid intima-media thickness, Metabolically healthy, Atherosclerosis

## Abstract

**Background:**

The relationship between osteocalcin and atherosclerosis remains unclear. This might be due to different degrees of confounding from factors that are associated with serum osteocalcin level, such as metabolic-related variables. This study aimed to investigate the relationship between serum osteocalcin level and carotid intima-media thickness (C-IMT) in a metabolically healthy population.

**Methods:**

A total of 476 subjects with normal values for weight, glucose tolerance, blood pressure, and lipids (age range, 20–75 years; 155 men, 201 premenopausal women, 120 postmenopausal women) from the Shanghai Obesity Study were recruited for this cross-sectional study. Subjects with a history of cardiovascular disease or carotid plaque were excluded. C-IMT was measured by ultrasonography. Serum osteocalcin level was assessed by an electrochemiluminescence immunoassay.

**Results:**

Median C-IMT in the entire study population was 0.55 mm with an interquartile range of 0.50–0.60 mm. C-IMT in premenopausal women was significantly lower than that in men and postmenopausal women (all *P* < 0.001). The median (interquartile range) of serum osteocalcin level in the entire population was 17.02 (13.31–21.47) ng/mL. Serum osteocalcin level in postmenopausal women was significantly higher than that in men and premenopausal women (all *P* < 0.001), while the level of serum osteocalcin in men was also significantly higher than that in premenopausal women (*P* < 0.001). No significant correlation was found between C-IMT and serum osteocalcin level in either men or postmenopausal women. There was a significant, inverse correlations between C-IMT and serum osteocalcin level in premenopausal women after adjustment of age, but this association was eliminated after adjustment for other confounding factors.

**Conclusions:**

Serum osteocalcin level was not independently associated with C-IMT in a metabolically healthy Chinese population.

## Introduction

Osteocalcin is a synthetic osteoblast-specific protein which is involved in the regulation of bone, glucolipid metabolism and in maintaining homeostasis [1–6]. Recent studies have investigated whether osteocalcin is associated with subclinical and clinical atherosclerotic coronary artery disease (CAD), but these studies have yielded inconsistent results. In some cases, studies have found inverse associations between osteocalcin and CAD [7–12]. For example, several studies have shown that subjects with CAD had significantly lower serum osteocalcin level than those without CAD [8, 9]. Similarly, serum osteocalcin level in subjects with type 2 diabetes mellitus (T2DM) has been found to be inversely correlated with subclinical measures of CAD, such as carotid intima-media thickness (C-IMT) and plaque score, independent of confounding factors [10–12].

In contrast to studies reporting an inverse association, some studies have reported no association or a positive association between serum osteocalcin level and measures of CAD. For example, population-based longitudinal study by Holvik et al. found that there was no association between plasma osteocalcin level and cardiovascular diseases (CVD) in younger-old subjects (65–74 years), but a higher plasma osteocalcin concentration was associated with a reduced risk of CVD in older-old men (≥75 years) while with an increased risk of CVD in older-old women (≥75 years) [13]. However, other research by Reyes-Garcia et al. observed that subjects with CAD had significantly higher serum osteocalcin level than those without CAD, and that serum osteocalcin level in women was positively correlated with C-IMT [14].

It is unclear why the studies noted above have reported contradictory findings on the association between serum osteocalcin level and atherosclerosis. The lack of consistency might be due to different study populations, or different degrees of confounding from factors that are associated with serum osteocalcin level, such as metabolic-related variables. In any event, the inconsistent results suggest that additional research into the association between osteocalcin and atherosclerosis is warranted, particularly in metabolically health populations that could help limit confounding from metabolic factors. Accordingly, the present study investigated the association between serum osteocalcin level and subclinical atherosclerosis in metabolically normal subjects without a history of CVD.

## Subjects and methods

### Subjects

Subjects were recruited from the Shanghai Obesity Study (SHOS) between December 2009 and December 2011. The detailed rules for implementation of the SHOS have been described in previous study [15]. Subjects were included if they met the following criteria for being normal-weight, metabolically healthy individuals: (i) body mass index (BMI) between at least 18.5 and less than 25.0 kg/m^2^ [16]; (ii) normal glucose tolerance, defined as a fasting plasma glucose (FPG) level less than 6.1 mmol/L and 2-h plasma glucose (2hPG) level less than 7.8 mmol/L [17]; (iii) normotensive, defined as systolic blood pressure (SBP) less than 130 mmHg and a diastolic blood pressure (DBP) less than 85 mmHg [18]; (iv) normal lipid status, defined as serum total cholesterol (TC) less than 5.18 mmol/L, serum triglycerides (TG) less than 1.70 mmol/L, serum high-density lipoprotein cholesterol (HDL-C) level of 1.04 mmol/L or higher, and serum low-density lipoprotein cholesterol (LDL-C) less than 3.37 mmol/L [19]. Subjects were excluded if they had a known history of CVD or carotid plaque, or had any infections, hepatic or renal dysfunction, malignant tumors, or were taking medicine that may influence the level of serum osteocalcin. After applying the inclusion and exclusion criteria noted above, a total of 476 subjects were available for study. The study was approved by the Ethics Committee of Shanghai Jiao Tong University Affiliated Sixth People’s Hospital, and was conducted in accordance with the Declaration of Helsinki. Data must be handled so as to not compromise study participants’ privacy. All participants provided written informed consent prior to enrollment.

### Questionnaire data

Each subject answered a standard questionnaire. The questionnaire assessed history of medical conditions, family history of disease, current medications use, and smoking history and other lifestyle factors. Subjects who smoked at least one cigarette per day for over half a year were defined as current smokers [15].

### Anthropometric and laboratory measurements

Height was measured with a stadiometer after the subject had removed his or her shoes and hats. Subjects were required to keep their eye-sight horizontal, with the back of the head, shoulder blades, buttocks, calves, and heels all touching the stadiometer’s vertical board, and their height was then recorded to the nearest 0.1 cm. Body weight was measured by a calibrated bathroom scale. Subjects removed their shoes and outer clothing and stood still in the middle of the scale, with feet slightly apart, until the scale’s weight reading had stabilized. Subjects’ weight was then recorded to the nearest 0.1 kg. BMI was calculated as body weight in kilo-grams divided by height in meters squared. Waist circumference (W) was measured at the horizontal plane between the inferior costal margin and the iliac crest on the mid-axillary line. Blood pressure was measured 3 times with a mercury sphygmomanometer at 3-min intervals, after the subject had been at rest for at lest 10 min. Form these three blood pressure measurements, a mean value was calculated. The techniques for laboratory measurement of blood samples have been described previously [15]. Serum total osteocalcin level was measured using an electrochemiluminescence immunoassay (Roche Diagnostics GmbH, Mannheim, Germany), and the intra-assay and inter-assay coefficients of variation for osteocalcin were 1.2 to 4.0 % and 1.7 to 6.5 %, respectively [15].

### Ultrasonographic measurement of C-IMT

An experienced radiologist, who was blinded to study results, made ultrasound measurements of C-IMT with a high-resolution B-mode scanner (Voluson 730 Expert; GE Healthcare, Waukesha, WI, USA). A 10-MHz probe was utilized, and C-IMT was measured on both sides of the common carotid artery approximately 1.0 cm proximal to the carotid bulb. The average C-IMT value of the two sides was used for analysis [20].

### Statistical analyses

Variables with normal distributions were described in terms of the mean ± standard deviation, while variables with skewed distribution were described in terms of the median and 25th and 75th percentiles. The independent-sample student’s *t*-test (for normally distributed variables) and the Wilcoxon rank-sum test (for skewed variables) were used to assess differences in characteristics between gender groups. Categorical variables were summarized using frequencies and percent, and differences in categorical variables among groups were assessed by the chi-square test. Partial correlations were calculated to analyze C-IMT’s association with serum osteocalcin and other clinical variables after adjustment for age. Multivariable linear regression models were run to examine the relationship between C-IMT (dependent variable) and serum osteocalcin level (independent variable) after adjustment for other variables. Statistical Product and Service Solutions software (SPSS for Windows, Version 16.0. Chicago, IL: SPSS Inc.) was used to perform statistical analyses. All statistical tests were two tailed, and p-values less than 0.05 were considered statistically significant.

## Results

### Clinical characteristics of study subjects

The 476 subjects enrolled in the present study had an age range of 20 to 75 years (mean 47.9 ± 9.5 years). Subjects were gender-stratified into 155 men, 201 premenopausal women, and 120 postmenopausal women. The years since menopause of postmenopausal women was 5.6 ± 4.6 years. The clinical characteristics of study subjects have been described in Table 1. Men had a significantly higher SBP, DBP level, and a higher frequency of smoking than either premenopausal or postmenopausal women (all *P* < 0.05). Postmenopausal women had higher levels of W, 2hPG, glycated hemoglobin (HbA1c), TC, TG, LDL-C, and C-reactive protein (CRP) than premenopausal women (all *P* < 0.05).

**Table 1:** Clinical characteristics of study subjects

Variables	Men	Premenopausal women	Postmenopausal women
n	155	201	120
Age (years)	50.1 ± 10.6	42.0 ± 6.8**	55.0 ± 4.4**^,^****
BMI (kg/m^2^)	22.0 ± 1.8	21.5 ± 1.7*	21.7 ± 1.8
W (cm)	78.0 (74.0–84.0)	72.0 (69.5–77.0)**	75.0 (71.0–80.0)**^,^****
SBP (mmHg)	119.3 (110.0–121.3)	110.7 (103.3–120.0)**	112.7 (107.7–120.0)*
DBP (mmHg)	74.7 (70.0–79.3)	72.0 (66.7–79.2)*	71.7 (69.3–78.7)*
FPG (mmol/L)	5.1 ± 0.4	5.1 ± 0.4	5.1 ± 0.4
2hPG (mmol/L)	5.5 ± 1.1	5.7 ± 1.0	6.0 ± 1.0**^,^****
HbA1c (%)	5.4 (5.2–5.6)	5.4 (5.1–5.6)	5.5 (5.3–5.8)**^,^****
FINS (mU/L)	5.5 (3.8–7.5)	6.4 (4.9–8.8)**	6.1 (4.5–7.6)
HOMA-IR	1.2 (0.8–1.7)	1.4 (1.1–2.0)**	1.4 (1.0–1.7)
TC (mmol/L)	4.34 ± 0.48	4.28 ± 0.54	4.54 ± 0.43**^,^****
TG (mmol/L)	0.88 (0.69–1.17)	0.79 (0.61–1.00)**	0.87 (0.65–1.16)***
HDL-C (mmol/L)	1.40 (1.25–1.61)	1.53 (1.37–1.78)**	1.62 (1.45–1.79)**
LDL-C (mmol/L)	2.65 ± 0.48	2.50 ± 0.46**	2.61 ± 0.44***
CRP (mg/L)	0.5 (0.3–0.8)	0.3 (0.2–0.7)**	0.5 (0.2–0.8)****
Smoking, n (%)	76 (49.0)	6 (3.0)**	1 (0.8)**
CVD family history, n (%)	45 (29.0)	52 (25.9)	47 (39.2)***

Data were presented as mean ± standard deviation or median (interquartile range)

*BMI* body mass index, *W* waist circumference, *SBP* systolic blood pressure, *DBP* diastolic blood pressure, *FPG* fasting plasma glucose, *2hPG* 2-h plasma glucose, *HbA1c* glycated hemoglobin, *FINS* fasting insulin, *HOMA-IR* homoeostasis model assessment-insulin resistance index, *TC* total cholesterol, *TG* triglyceride, *HDL-C* high-density lipoprotein cholesterol, *LDL-C* low-density lipoprotein cholesterol, *CRP* C-reactive protein, *CVD* cardiovascular disease

*P < 0.05; **P < 0.01 vs. men; ***P < 0.05; ****P < 0.01 vs. premenopausal women

### Comparison of C-IMT and serum osteocalcin level according to gender categorizes

The median C-IMT for the total study population was 0.55 mm with an interquartile range of 0.50–0.60 mm. The C-IMT of premenopausal women was significantly lower than that of men [0.50 (0.50–0.60) mm vs. 0.60 (0.50–0.65) mm, *P* < 0.001] and postmenopausal women [0.50 (0.50–0.60) mm vs. 0.60 (0.55–0.60) mm, *P* < 0.001]. However, the difference in C-IMT between men and postmenopausal women was not statistically significant [0.60 (0.50–0.65) mm vs. 0.60 (0.55–0.60) mm, *P* > 0.05].

**Fig. 1:**
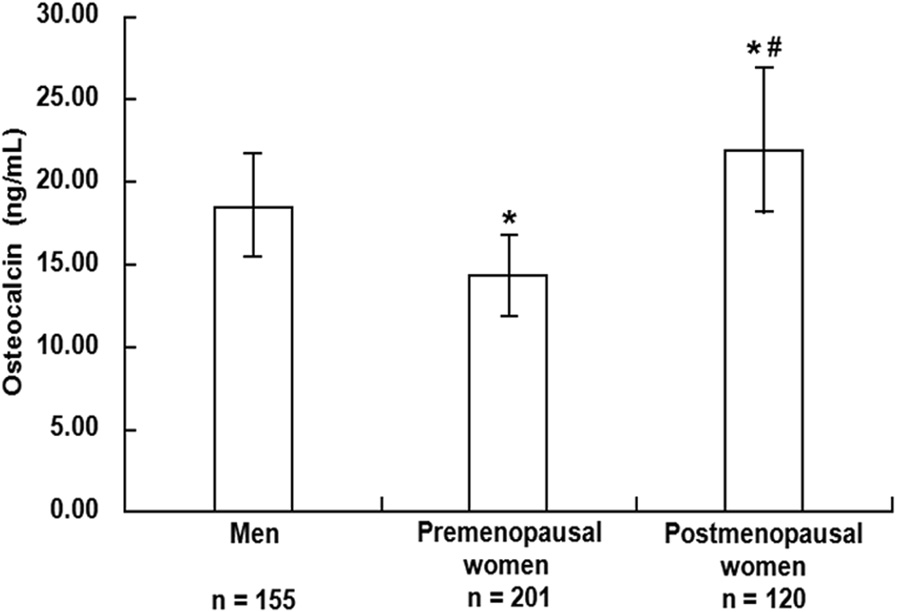
Serum osteocalcin level in subjects with different gender categorizes. Data were shown as median with 25th and 75th percentiles. *P < 0.001 compared with men, ^#^ *P* < 0.001 compared with premenopausal women

The overall median (interquartile range) serum osteocalcin level was 17.02 (13.31–21.47) ng/mL, with a level of 18.48 (15.53–21.74) ng/mL in men, 14.36 (11.88–16.79) ng/mL in premenopausal women, and 21.86 (18.15–26.90) ng/mL in postmenopausal women. Serum osteocalcin level in postmenopausal women was significantly higher than in men and premenopausal women (all *P* < 0.001), while serum osteocalcin level in men was also significantly higher than in premenopausal women (*P* < 0.001) (Fig. 1).

### Association of C-IMT with other anthropometric and biochemical parameters

As shown in Table 2, the correlation between C-IMT and serum osteocalcin level was not significant in men or postmenopausal women after adjustment of age (all *P* > 0.05). However, in premenopausal women, C-IMT showed a significant, inverse correlation with serum osteocalcin level (*r* = −0.145, *P* = 0.041). Therefore, a stepwise multivariable regression model was run only in premenopausal women to determine if serum osteocalcin level was associated with C-IMT in these subjects, independent of other confounding factors. C-IMT was the dependent variable, and the parameters significantly correlated with C-IMT were set as independent variables, including age, BMI, W, SBP, DBP, FPG, serum fasting insulin (FINS), CRP, smoking status, and CVD family history, and osteocalcin. The model indicated that age, SBP, FPG, FINS and CRP were independently associated with C-IMT (all *P* < 0.05, Table 3), but that serum osteocalcin level was not independently associated with C-IMT in premenopausal women (*P* >0.05).

**Table 2:** Correlations of C-IMT with various clinical and biochemical parameters after adjustment for age

Variables	Men		Premenopausal women		Postmenopausal women	
	*r*	*P*	*r*	*P*	*r*	*P*
BMI	0.067	0.410	0.162	0.022	−0.027	0.768
W	0.140	0.083	0.153	0.030	−0.002	0.982
SBP	0.269	0.001	0.205	0.004	0.194	0.035
DBP	0.140	0.083	0.168	0.017	0.108	0.243
FPG	0.101	0.212	0.232	0.001	0.024	0.793
2hPG	−0.090	0.264	−0.046	0.519	−0.020	0.827
HbA1c	−0.137	0.091	−0.054	0.447	0.099	0.286
FINS	0.267	0.001	0.262	<0.001	0.103	0.267
HOMA-IR	0.269	0.001	0.283	<0.001	0.103	0.264
TC	−0.141	0.081	−0.034	0.630	−0.060	0.518
TG	0.137	0.090	0.087	0.220	−0.003	0.974
HDL-C	−0.219	0.006	−0.046	0.518	0.035	0.709
LDL-C	0.113	0.161	0.089	0.209	0.062	0.502
CRP	0.011	0.889	0.208	0.003	0.141	0.126
Osteocalcin	0.034	0.674	−0.145	0.041	−0.156	0.090

*C-IMT* carotid intima-media thickness, *BMI* body mass index, *W* waist circumference, *SBP* systolic blood pressure, *DBP* diastolic blood pressure, *FPG* fasting plasma glucose, *2hPG* 2-h plasma glucose, *HbA1c* glycated hemoglobin, *FINS* fasting insulin, *HOMA-IR* homoeostasis model assessment-insulin resistance index, *TC* total cholesterol, *TG* triglyceride, *HDL-C* high-density lipoprotein cholesterol, *LDL-C* low-density lipoprotein cholesterol, *CRP* C-reactive protein

**Table 3:** Association of C-IMT with other anthropometric and biochemical parameters in premenopausal women

Independent variables	Standardized *β*	*t*	*P*
Age	0.300	4.605	<0.001
SBP	0.135	1.986	0.048
FPG	0.150	2.345	0.020
FINS	0.176	2.684	0.008
CRP	0.209	3.423	0.001

Independent variables included in premenopausal women were as follows: age, BMI, W, SBP, DBP, FPG, FINS, CRP, smoking status, CVD family history, and osteocalcin. Only the statistical significant parameters correlated with C-IMT were presented in Table 3 after stepwise regression analysis

*C-IMT* carotid intima-media thickness, *BMI* body mass index, *W* waist circumference, *SBP* systolic blood pressure, *DBP* diastolic blood pressure, *FPG* fasting plasma glucose, *FINS* fasting insulin, *CRP* C-reactive protein, *CVD* cardiovascular disease

## Discussion

Evidences indicate that skeleton works not only as a structural scaffold but also as an endocrine organ. Many bioactive factors secreted from bone, such as osteocalcin and osteoprotegerin, have a regulatory role in energy metabolism [21, 22]. Recent studies have suggested a potentially important role for osteocalcin in regulating the function of vascular endothelial cells, and the cardiovascular system [23, 24]. This suggests osteocalcin could be involved in the development of CAD. Several studies have investigated the association between osteocalcin and atherosclerosis, but results have not been consistent. Some studies suggest osteocalcin may actually be protective against early atherosclerosis. For example, a study from Korea demonstrated an independent effect of osteocalcin on vascular endothelial cells, suggesting that osteocalcin could have beneficial effects on atherosclerosis [25]. Our previous study in high fat diet animals indicate that osteocalcin has an endothelial-protective effect in atherosclerosis through mediating the PI3K/Akt/eNOS signaling pathway, and exogenous osteocalcin can improve the function of human umbilical vein endothelial cells *in vitro* [26]. Consistent with this result, a study in 1319 postmenopausal women found serum osteocalcin level was independently and inversely correlated with C-IMT [27].

The arteries plaques and aortic calcifications would emerge in the development and progression of atherosclerosis. Moreover, coronary atherosclerosis is the major adverse cardiac events of atherosclerosis. Likewise, a study from Japan of 50 patients with T2DM found that changes in the serum osteocalcin level were significantly and inversely correlated with plaque score [12]. Another study of 774 elderly men found that higher osteocalcin level was associated with lower abdominal aortic calcification progression [28]. Additionally, our group and others have found inverse associations between serum osteocalcin level and coronary atherosclerosis in Chinese men [8, 9].

However, in contrast to the studies above, some data indicate that osteocalcin shows no association, or even shows a positive association, with atherosclerosis or CVD. Thus, a follow-up study of 1290 Korean men found that the serum osteocalcin level was not associated with the development of CVD, even after adjustment for other risk factors for CVD [29]. Another 4.1-year follow-up study also reported no association between plasma osteocalcin level and CVD in younger-old subjects (65–74 years) [13]. A positive correlation between serum osteocalcin level and atherosclerosis was found in a study of 78 patients with T2DM. In particular, the level of osteocalcin in subjects with CAD was higher than in those without CAD, and higher concentrations of osteocalcin were found in women with abnormal IMT, carotid plaques, and aortic calcifications compared to normal women [14].

It is unclear why the results noted above have been so inconsistent. One possible explanation is that the studies were conducted in different populations and ethnic groups. Another explanation may be that different studies were affected to different degrees by confounding from metabolic factors. Studies have demonstrated there is a close correlations between serum osteocalcin level and glucolipid metabolic disorders, obesity, and metabolic syndrome. Each of these metabolic dysfunctions are related to the progression of atherosclerosis, and therefore, are considered as risk factors for CVD [30]. Researches indicate that subjects with diabetes or impaired glucose tolerance have significantly lower serum osteocalcin level than the subjects with normal glucose tolerance [31, 32], and that serum osteocalcin level of subjects with metabolic syndrome are lower than in those without metabolic syndrome [33]. These findings suggest that the association of osteocalcin with atherosclerosis may be influenced by metabolic variables. Thus, to eliminate potential confounding from metabolic variables, it might be useful to explore the relationship between serum osteocalcin level and atherosclerosis in metabolically healthy persons. In this regard, a study of 638 men with normal glucose tolerance by Ma et al. showed an inverse association between serum osteocalcin level and the prevalence of carotid plaque. However, the study did not account for the influence of other metabolic factors, such as obesity, hypertension and dyslipidemia [31]. Therefore, to better clarify the relationship of serum osteocalcin level to atherosclerosis without confounding from metabolic variables, the present study excluded subjects with traditional risk factors for CVD (overweight, obesity, hyperglycemia, hypertension, and dyslipidemia), and further eliminated those with history of CVD or carotid plaque. In accordance with the previous findings of gender-related difference in serum osteocalcin concentration [32], the serum osteocalcin level of postmenopausal women was significantly higher than those of men and premenopausal women in this study. There was no statistically significant correlation between serum osteocalcin level and C-IMT in either men or postmenopausal women. Although an simple correlation between these two factors was found in premenopausal women, a multivariable model failed to demonstrate a significant association between serum osteocalcin level and C-IMT after adjusting for relevant confounding factors. Our previous study involving animal models also found that exogenous osteocalcin was not related to endothelium-dependent relaxation in mice fed with chow diet, which is in agreement with the present study’s finding of metabolically healthy human to some extent [26].

### Limitations

Because of the cross-sectional nature of this study, we were not able to clarify whether the association between osteocalcin and C-IMT was causal. Thus, additional prospective studies are needed to assess this association. In addition, serum total osteocalcin is composed of uncarboxylated and carboxylated forms. Initial animal and *in vitro* studies indicated that undercarboxylated osteocalcin is the biologically active isoform mediating the metabolic functions. However, several recent clinical studies have demonstrated that not only undercarboxylated but also total osteocalcin were associated with energy metabolism and atherosclerosis as well [8, 9, 14]. Because lack of an automated assay to examine the uncarboxylated form, our study only measured serum total osteocalcin.

## Conclusions

This study is the first to report that serum osteocalcin level is not independently correlated with C-IMT in a metabolically healthy population.

## Abbreviations

BMI: Body mass index
CAD: Coronary artery disease
C-IMT: Carotid intima-media thickness
CRP: C-reactive protein
CVD: Cardiovascular disease
DBP: Diastolic blood pressure
FINS: Fasting insulin
FPG: Fasting plasma glucose
HbA1c: Glycated hemoglobin
HDL-C: High-density lipoprotein cholesterol
HOMA-IR: Homoeostasis model assessment-insulin resistance index
LDL-C: Low-density lipoprotein cholesterol
SBP: Systolic blood pressure
SHOS: Shanghai Obesity Study
T2DM: Type 2 diabetes mellitus
TC: Total cholesterol
TG: Triglyceride
W: Waist circumference
2hPG: 2-h plasma glucose

## References

[1] Szulc P, Seeman E, Delmas PD (2000). Biochemical measurements of bone turnover in children and adolescents. Osteoporos Int.

[2] Díaz-López A, Bulló M, Juanola-Falgarona M, Martínez-González MA, Estruch R, Covas MI (2013). Reduced serum concentrations of carboxylated and undercarboxylated osteocalcin are associated with risk of developing type 2 diabetes mellitus in a high cardiovascular risk population: a nested case–control study. J Clin Endocrinol Metab.

[3] Ducy P (2011). The role of osteocalcin in the endocrine cross-talk between bone remodelling and energy metabolism. Diabetologia.

[4] Movahed A, Larijani B, Nabipour I, Kalantarhormozi M, Asadipooya K, Vahdat K (2012). Reduced serum osteocalcin concentrations are associated with type 2 diabetes mellitus and the metabolic syndrome components in postmenopausal women: the crosstalk between bone and energy metabolism. J Bone Miner Metab.

[5] Oosterwerff MM, van Schoor NM, Lips P, Eekhoff EM (2013). Osteocalcin as a predictor of the metabolic syndrome in older persons: a population-based study. Clin Endocrinol (Oxf).

[6] Prats-Puig A, Osiniri I, Soriano-Rodríguez P, Carreras-Badosa G, Buñuel-Álvarez JC, Vila-Pablos C (2014). Undercarboxylated osteocalcin relates to cardiovascular risk markers in offspring of families with metabolic syndrome. Atherosclerosis.

[7] Yeap BB, Chubb SA, Flicker L, McCaul KA, Ebeling PR, Hankey GJ (2012). Associations of total osteocalcin with all-cause and cardiovascular mortality in older men. The health in men study. Osteoporos Int..

[8] Bao Y, Zhou M, Lu Z, Li H, Wang Y, Sun L (2011). Serum levels of osteocalcin are inversely associated with the metabolic syndrome and the severity of coronary artery disease in Chinese men. Clin Endocrinol (Oxf).

[9] Zhang Y, Qi L, Gu W, Yan Q, Dai M, Shi J (2010). Relation of serum osteocalcin level to risk of coronary heart disease in Chinese adults. Am J Cardiol.

[10] Sheng L, Cao W, Cha B, Chen Z, Wang F, Liu J (2013). Serum osteocalcin level and its association with carotid atherosclerosis in patients with type 2 diabetes. Cardiovasc Diabetol..

[11] Kanazawa I, Yamaguchi T, Yamamoto M, Yamauchi M, Kurioka S, Yano S (2009). Serum osteocalcin level is associated with glucose metabolism and atherosclerosis parameters in type 2 diabetes mellitus. J Clin Endocrinol Metab.

[12] Kanazawa I, Yamaguchi T, Sugimoto T (2011). Relationship between bone biochemical markers versus glucose/lipid metabolism and atherosclerosis; a longitudinal study in type 2 diabetes mellitus. Diabetes Res Clin Pract.

[13] Holvik K, van Schoor NM, Eekhoff EM, den Heijer M, Deeg DJ, Lips P (2014). Plasma osteocalcin levels as a predictor for cardiovascular disease in older men and women: A population-based cohort study. Eur J Endocrinol.

[14] Reyes-Garcia R, Rozas-Moreno P, Jimenez-Moleon JJ, Villoslada MJ, Garcia-Salcedo JA, Santana-Morales S (2012). Relationship between serum levels of osteocalcin and atherosclerotic disease in type 2 diabetes. Diabetes Metab.

[15] Bao Y, Ma X, Yang R, Wang F, Hao Y, Dou J (2013). Inverse relationship between serum osteocalcin levels and visceral fat area in Chinese men. Inverse relationship between serum osteocalcin levels and visceral fat area in Chinese men. J Clin Endocrinol Metab..

[16] World Health Organization (2000). Obesity: Preventing and managing the global epidemic. Report of a WHO consultation. World Health Organ Tech Rep Ser.

[17] Department of Noncommunicable Disease Surveillance (1999). Report of a WHO consultation: definition, diagnosis and classification of diabetes mellitus and its complication. Part 1: Diagnosis and classification of diabetes mellitus.

[18] Peleska J, Zvara K, Vesely A, Zvarova J, Tomeckova M, Hanzlicek P (2002). Development of electronic form of the 1999 WHO/ISH hypertension guidelines. Stud Health Technol Inform..

[19] Joint Committee for Developing Chinese Guidelines on Prevention and Treatment of Dyslipidemia in Adults (2007). Chinese guidelines on prevention and treatment of dyslipidemia in adults. Zhonghua Xin Xue Guan Bing Za Zhi.

[20] Hao Y, Ma X, Luo Y, Ni J, Dou J, Zhu J (2014). Additional role of serum 25-hydroxyvitamin D(3) levels in atherosclerosis in Chinese middle-aged and elderly men. Clin Exp Pharmacol Physiol.

[21] Maser RE, Lenhard MJ, Sneider MB, Pohlig RT (2015). Osteoprotegerin is a better serum biomarker of coronary artery calcification than osteocalcin in type 2 diabetes. Endocr Pract.

[22] Aoki A, Murata M, Asano T, Ikoma A, Sasaki M, Saito T (2013). Association of serum osteoprotegerin with vascular calcification in patients with type 2 diabetes. Cardiovasc Diabetol..

[23] Gössl M, Mödder UI, Atkinson EJ, Lerman A, Khosla S (2008). Osteocalcin expression by circulating endothelial progenitor cells in patients with coronary atherosclerosis. J Am Coll Cardiol.

[24] Bini A, Mann KG, Kudryk BJ, Schoen FJ (1999). Noncollagenous bone matrix proteins, calcification, and thrombosis in carotid artery atherosclerosis. Arterioscler Thromb Vasc Biol.

[25] Jung CH, Lee WJ, Hwang JY, Lee MJ, Seol SM, Kim YM (2013). The preventive effect of uncarboxylated osteocalcin against free fatty acid-induced endothelial apoptosis through the activation of phosphatidylinositol 3-kinase/Akt signaling pathway. Metabolism.

[26] Dou J, Li H, Ma X, Zhang M, Fang Q, Nie M (2014). Osteocalcin attenuates high fat diet-induced impairment of endothelium-dependent relaxation through Akt/eNOS-dependent pathway. Cardiovasc Diabetol..

[27] Yang R, Ma X, Dou J, Wang F, Luo Y, Li D (2013). Relationship between serum osteocalcin levels and carotid intima-media thickness in Chinese postmenopausal women. Menopause.

[28] Confavreux CB, Szulc P, Casey R, Boutroy S, Varennes A, Vilayphiou N (2013). Higher serum osteocalcin is associated with lower abdominal aortic calcification progression and longer 10-year survival in elderly men of the MINOS cohort. J Clin Endocrinol Metab.

[29] Hwang YC, Kang M, Cho IJ, Jeong IK, Ahn KJ, Chung HY (2015). Association between the circulating OCN level and the development of cardiovascular disease in middle-aged men: a mean 8.7-year longitudinal follow-up study. J Atheroscler Thromb.

[30] Ferreira I, Boreham CA, Twisk JW, Gallagher AM, Young IS, Murray LJ (2007). Clustering of metabolic syndrome risk factors and arterial stiffness in young adults: the Northern Ireland Young Hearts Project. J Hypertens.

[31] Ma H, Lin H, Hu Y, Li X, He W, Jin X (2014). Serum levels of osteocalcin in relation to glucose metabolism and carotid atherosclerosis in Chinese middle-aged and elderly male adults: The Shanghai Changfeng Study. Eur J Intern Med.

[32] Zhou M, Ma X, Li H, Pan X, Tang J, Gao Y (2009). Serum osteocalcin concentrations in relation to glucose and lipid metabolism in Chinese individuals. Eur J Endocrinol.

[33] Yang R, Ma X, Pan X, Wang F, Luo Y, Gu C (2013). Serum osteocalcin levels in relation to metabolic syndrome in Chinese postmenopausal women. Menopause.

